# Linear association between atherogenic index of plasma and diabetic kidney disease: evidence from a large clinical cohort and NHANES

**DOI:** 10.3389/fnut.2026.1853881

**Published:** 2026-08-11

**Authors:** Jinghan Zheng, Jingwei Chi, Kui Che, Yangang Wang, Qidong Zheng

**Affiliations:** 1 Department of Endocrinology, Affiliated Hospital of Qingdao University, Qingdao, Shandong, China; 2 Department of Internal Medicine, Yuhuan Second People’s Hospital, Yuhuan, Zhejiang, China

**Keywords:** atherogenic index of plasma, cross-sectional study, diabetic kidney disease, linear association, type 2 diabetes

## Abstract

**Background:**

Diabetic kidney disease (DKD) is a prevalent microvascular complication of type 2 diabetes mellitus (T2DM) and contributes substantially to end-stage renal disease. The atherogenic index of plasma (AIP) has exhibited biomarker utility. However, its relationship with DKD remains uncertain.

**Methods:**

This cross-sectional investigation included 10,112 T2DM subjects from the National Metabolic Management Center of Yuhuan Second People’s Hospital between September 2017 and February 2025 and 5,573 individuals in NHANES cohort (1999–2020). AIP values were calculated and categorized into quartiles. Multivariable logistic regression, restricted cubic spline, and stratified subgroup analyses were applied for assessing the link between AIP and DKD. The area under the curve (AUC), net reclassification improvement (NRI) and integrated discrimination improvement (IDI) was used to assess the discriminative ability of AIP.

**Results:**

After adjustment for confounding factors, each one-unit AIP rise resulted in a 72% higher likelihood of DKD (OR: 1.72, 95% CI: 1.51–1.96, *p* < 0.001). Relative to the lowest AIP quartile, the highest quartile showed a 54% higher odds DKD (OR: 1.54, 95% CI: 1.36–1.75, *p* < 0.001), with a clear dose–response effect (P for trend <0.001). Spline modeling indicated a positive linear link between AIP and DKD, which was apparent in all examined subgroups (all P for interaction >0.05) and externally validated in NHANES. Incorporating AIP into the baseline model modestly improved discrimination, increasing the area under the curve from 68.42 to 68.76%, with a continuous NRI of 0.118 (95% CI: 0.079–0.157, *p* < 0.001) and an IDI of 0.006 (95% CI: 0.004–0.007, *p* < 0.001).

**Conclusion:**

AIP shows an independent positive association with DKD in individuals with T2DM, demonstrating a linear dose–response relationship, but the incremental predictive value is limited. The association between AIP and DKD still needs to be further validated by larger-scale prospective studies.

## 1 Introduction

Type 2 diabetes mellitus (T2DM) is a continuing threat to global public health, with 529 million individuals affected in 2021 and up to 1.31 billion by 2050 (1). It is associated with many chronic complications, particularly diabetic kidney disease (DKD), which occurs in 20–40% of diabetic patients over their lifetime (2). DKD presents with albuminuria, estimated glomerular filtration rate (eGFR) values in progressive decline, and a greater likelihood of cardiovascular disorders and death from all causes (3). Even with recent improvements in glycemic and blood pressure management, DKD remains a major predecessor of end-stage renal disease and frequently necessitates dialysis (4). This substantial burden highlights the need for effective biomarkers that can facilitate early identification and risk stratification (5).

Abnormal lipid metabolism is recognized as a key contributor to DKD pathogenesis, promoting inflammation, endothelial dysfunction, and oxidative stress (6). The atherogenic index of plasma (AIP), originally introduced in 2007 based on the work of Dobiásová and Frohlich (7), is computed as the logarithm of the ratio of triglycerides to high-density lipoprotein cholesterol. Compared with conventional lipid measures, AIP is reflective of both atherogenic and protective lipids and serves as an indirect indicator of low-density lipoproteins (8). A growing abundance of evidence suggests that AIP is related to insulin resistance and other forms of metabolic abnormalities, including metabolic syndrome and cardiovascular disease risk (9, 10). Numerous studies have examined the relationship between AIP and renal disease in diabetes, with many reporting a positive association between elevated AIP and DKD (11–14). However, not all findings are consistent, as some investigations have failed to demonstrate a significant link between AIP and specific renal endpoints (15). These inconsistencies may be related to different populations, definitions of renal outcomes, and the extent of confounder adjustment. In addition, many prior studies have evaluated albuminuria or reduced eGFR separately rather than applying a comprehensive definition of DKD (16, 17).

In light of these gaps in knowledge, the present large-scale cross-sectional study incorporating 10,112 subjects with T2DM was designed to systematically explore how AIP is associated with DKD prevalence. To further strengthen the generalizability of our findings, data from the National Health and Nutrition Examination Survey (NHANES) were additionally obtained as an external validation cohort.

## 2 Materials and methods

### 2.1 Study participants

**Figure 1:**
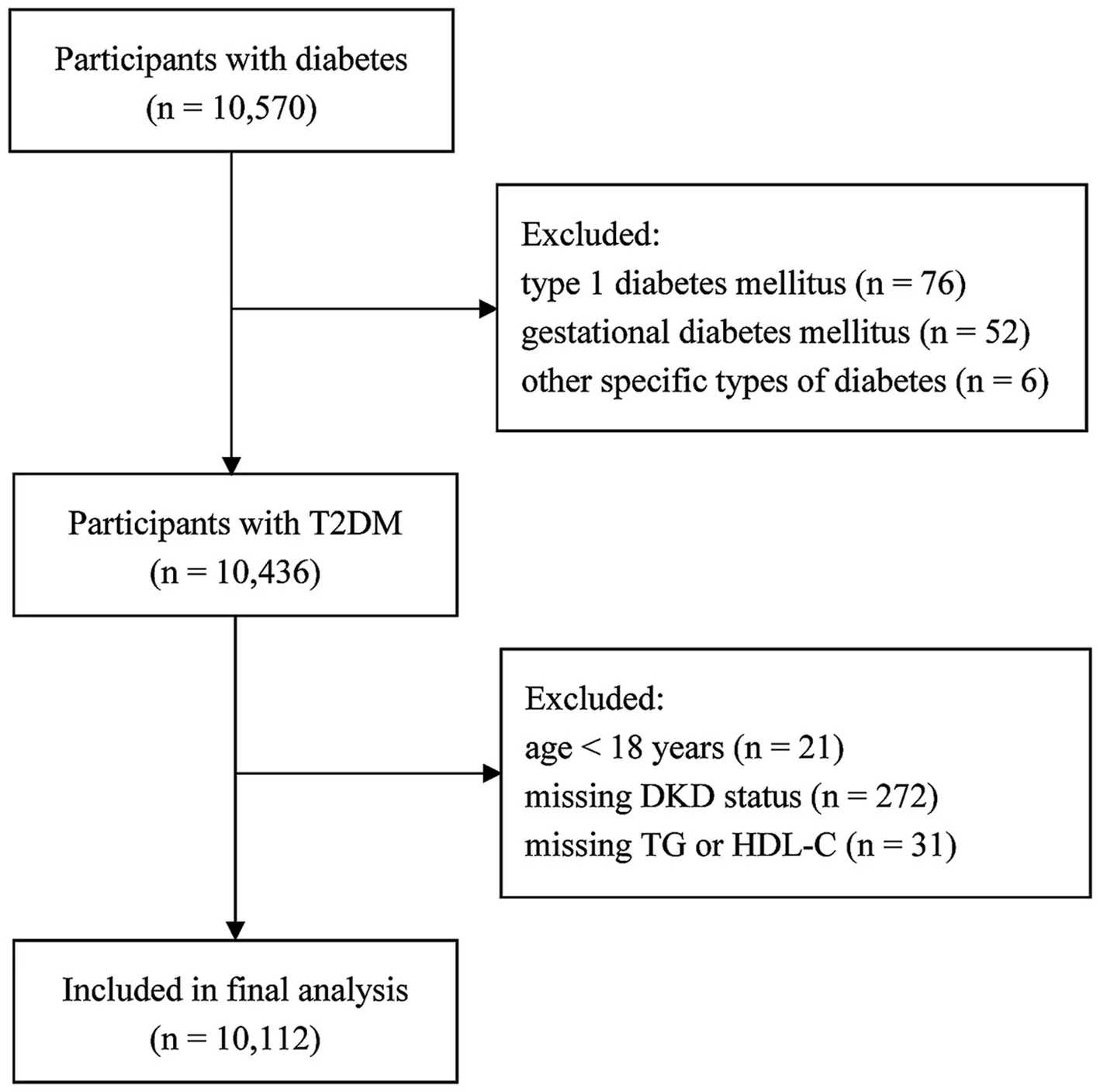
Flowchart of the study.

This cross-sectional investigation was undertaken at the National Metabolic Management Center (MMC) of Yuhuan Second People’s Hospital, following approval by the institutional Clinical Research Ethics Committee, with all subjects offering written consent as per the tenats of the Declaration of Helsinki. In total, 10,570 individuals with diabetes were initially screened between September 2017 and February 2025. Of these, those non-T2DM (including type 1 diabetes mellitus, gestational diabetes mellitus, and other specific types of diabetes), below 18 years old, lacking information on DKD status, or with incomplete TG or HDL-C measurements were excluded. After applying these criteria, 10,112 individuals were retained for analysis (Figure 1).

All NHANES protocols were approved by the NCHS Research Ethics Review Board, with all subjects having given written informed consent. This analysis combined information from 10 NHANES cycles between 1999 and 2020, for an initial sample of 107,622 individuals. After screening participants according to the prespecified exclusion criteria (cases lacking DKD information or with incomplete measurements of TG and HDL-C), 5,573 individuals were included in the final analytic sample.

### 2.2 Data collection

Data were obtained from the MMC database, including demographic variables (age, sex, education level), anthropometric measurements (BMI, systolic and diastolic blood pressure [SBP, DBP]), glycemic indices (fasting blood glucose [FBG], postprandial blood glucose [PBG], glycated hemoglobin [HbA1c], fasting and postprandial C-peptide [FCp, PCp]), lipid parameters (total cholesterol [TC], TG, HDL-C, low-density lipoprotein cholesterol [LDL-C]), renal function indicators (eGFR, uric acid [UA], serum creatinine [Scr], blood urea nitrogen [BUN], urinary albumin-to-creatinine ratio [UACR]), liver enzymes (ALT, AST), comorbid conditions (hypertension, dyslipidemia), lifestyles (smoking and drinking), and medications (antihypertensive and lipid-lowering therapies). BMI represents weight (kg) divided by the square of height (m^2^). AIP was derived as the logarithm of the TG-to-HDL-C ratio. eGFR calculations made use of the Chronic Kidney Disease Epidemiology Collaboration equation (18). DKD was defined per ADA guidelines (19), as the either UACR ≥30 mg/g or eGFR <60 mL/min/1.73 m^2^.

Covariate data were obtained from NHANES interviews such as: age, sex, DBP, SBP, BMI, lipid-lowering medication, and antihypertensive medication. Key laboratory measures included FBG, TG, TC, HDL-C, LDL-C, HbA1c, UACR, and eGFR.

### 2.3 Statistical analyses

Normally distributed continuous data are shown as means ± standard deviation (SD) and skewed data are presented as medians with interquartile range (IQR). Categorical variables are given as frequencies and percentages. Patients were stratified into quartiles according to AIP values (Q1–Q4). Differences across groups were assessed using one-way ANOVA and Kruskal-Wallis and chi-square tests, as appropriate. Under 10% of covariate data were missing (Supplementary Table 1). Multiple imputation using five iterations was employed when addressing missing values. Four models were sequentially constructed: Model 1 (not adjusted), Model 2 (adjusted for sex and age), Model 3 (further adjusted for SBP, DBP, FBG, HbA1c, and FCp), and Model 4 (further including BMI and medication use). To further evaluate multicollinearity, variance inflation factor (VIF) values were calculated for all covariates. All the covariates have no multicollinearity (Supplementary Table 2). AIP was utilized as both a continuous variable and as quartiles, with the lowest quartile used as reference. Dose–response relationships were assessed using restricted cubic spline (RCS) regression with knots at the 5th, 35th, 65th, and 95th percentiles of the AIP distribution. Subgroup analyses were performed to evaluate the consistency of the AIP-DKD association across key clinical strata, including age (≥ 60, <60 years), sex (male, female), BMI (<24, ≥24–< 28, ≥28 kg/m^2^), diabetes duration (<5, ≥5–< 10, ≥10 years), HbA1c (<7%, ≥7%–< 9%, ≥9%), hypertension (yes vs. no), and dyslipidemia (yes vs. no). Heterogeneity was assessed by incorporation of interaction terms into regression models. The area under the curve (AUC), continuous net reclassification improvement (NRI), and integrated discrimination improvement (IDI) values were utilized to compare the predictive utility of the baseline model with and without AIP. Data were analyzed using R v4.2.2, with a two-sided *p* < 0.05 representing significance.

## 3 Results

### 3.1 Baseline participant information

The study cohort included 10,112 T2DM cases who were categorized into AIP quartiles. The highest quartile (Q4) included a predominance of males (68.0% vs. 50.0%, *p* < 0.001), as well as, on average, younger individuals compared to the lowest quartile (52.7 ± 12.7 vs. 58.6 ± 10.8 years, *p* < 0.001). Increasing AIP levels were associated with less favorable metabolic profiles, including poorer glycemic control and dyslipidemia corresponding to elevated triglycerides and reduced HDL-C (all *p* < 0.001). The proportion of patients with DKD rose from 39.3% in Q1 to 52.9% in Q4 (*p* < 0.001). Participant information is detailed in Table 1.

**Table 1:** Baseline characteristics of study participants according to AIP quartiles.

Characteristics	Total (*n* = 10,112)	Q1 (*n* = 2,527)	Q2 (*n* = 2,529)	Q3 (*n* = 2,528)	Q4 (*n* = 2,528)	*p* value
Demographic characteristics						
Male, *n* (%)	5,737 (56.7)	1,264 (50.0)	1,307 (51.7)	1,446 (57.2)	1,720 (68.0)	<0.001
Age, years	56.3 ± 11.9	58.6 ± 10.8	57.4 ± 11.4	56.3 ± 11.9	52.7 ± 12.7	<0.001
High school education or above, *n* (%)	932 (9.2)	159 (6.3)	191 (7.6)	263 (10.4)	319 (12.6)	<0.001
Anthropometric and vital parameters						
BMI, kg/m^2^	25.6 ± 3.7	24.1 ± 3.4	25.5 ± 3.6	26.1 ± 3.7	26.6 ± 3.6	<0.001
SBP, mm Hg	131.7 ± 18.4	129.9 ± 19.3	131.9 ± 18.5	131.9 ± 18.2	133.1 ± 17.6	<0.001
DBP, mm Hg	74.8 ± 11.4	72.0 ± 11.0	74.2 ± 10.8	75.3 ± 11.5	77.6 ± 11.4	<0.001
Glycemic parameters						
Diabetes duration, years	2.9 (0.2, 9.8)	3.9 (0.5, 11.1)	3.2 (0.2, 10.3)	2.8 (0.1, 9.6)	2.0 (0.0, 6.7)	<0.001
FBG, mmol/L	9.3 ± 4.2	8.8 ± 3.9	8.9 ± 3.8	9.2 ± 3.8	10.4 ± 4.9	<0.001
PBG, mmol/L	14.9 ± 6.2	14.6 ± 6.5	14.6 ± 6.0	14.7 ± 5.9	15.9 ± 6.2	<0.001
HbA1c, %	8.5 ± 2.2	8.2 ± 2.3	8.3 ± 2.2	8.4 ± 2.1	8.9 ± 2.3	<0.001
FCp, ng/mL	2.3 (1.7, 3.0)	1.7 (1.3, 2.3)	2.2 (1.6, 2.9)	2.4 (1.9, 3.2)	2.8 (2.2, 3.7)	<0.001
PCp, ng/mL	5.2 (3.5, 7.5)	4.4 (2.8, 6.4)	5.2 (3.5, 7.4)	5.5 (3.8, 7.9)	5.8 (4.1, 8.3)	<0.001
Lipid profile						
TG, mmol/L	1.5 (1.0, 2.2)	0.8 (0.7, 1.0)	1.2 (1.1, 1.4)	1.8 (1.5, 2.1)	3.1 (2.5, 4.5)	<0.001
TC, mmol/L	5.1 (4.3, 6.0)	4.9 (4.1, 5.7)	5.0 (4.3, 5.8)	5.2 (4.4, 6.0)	5.5 (4.6, 6.3)	<0.001
HDL-C, mmol/L	1.1 (0.9, 1.4)	1.4 (1.2, 1.7)	1.2 (1.1, 1.4)	1.1 (0.9, 1.2)	0.9 (0.8, 1.0)	<0.001
LDL-C, mmol/L	3.0 ± 1.0	2.8 ± 1.0	3.1 ± 1.0	3.1 ± 1.0	2.8 ± 1.1	<0.001
Renal parameters						
BUN, mmol/L	5.4 (4.5, 6.6)	5.6 (4.6, 6.7)	5.4 (4.5, 6.6)	5.4 (4.4, 6.6)	5.3 (4.4, 6.5)	<0.001
Scr, μmol/L	62.0 (51.0, 75.0)	59.0 (49.0, 72.0)	62.0 (50.0, 75.0)	63.0 (52.0, 77.0)	65.0 (53.0, 78.0)	<0.001
UA, μmol/L	338.2 ± 101.2	300.0 ± 86.9	327.6 ± 90.7	346.8 ± 96.6	378.5 ± 112.2	<0.001
DKD, *n* (%)	4,597 (45.5)	992 (39.3)	1,086 (42.9)	1,182 (46.8)	1,337 (52.9)	<0.001
Liver parameters						
ALT, U/L	22.0 (16.0, 35.0)	19.0 (14.0, 28.0)	21.0 (15.0, 33.0)	24.0 (17.0, 37.0)	28.0 (18.0, 45.0)	<0.001
AST, U/L	19.0 (16.0, 26.0)	19.0 (15.0, 24.0)	19.0 (15.0, 25.0)	20.0 (16.0, 27.0)	21.0 (16.0, 29.0)	<0.001
Comorbidities and lifestyle factors						
Hypertension, *n* (%)	5,893 (58.3)	1,317 (52.1)	1,506 (59.5)	1,538 (60.8)	1,532 (60.6)	<0.001
Dyslipidemia, *n* (%)	6,716 (66.4)	1,026 (40.6)	1,285 (50.8)	1,925 (76.1)	2,480 (98.1)	<0.001
Smoking, *n* (%)	2,458 (24.3)	419 (16.6)	528 (20.9)	669 (26.5)	842 (33.3)	<0.001
Alcohol consumption, *n* (%)	1,294 (12.8)	273 (10.8)	282 (11.2)	310 (12.3)	429 (17.0)	<0.001
Medications						
Antihypertensive medication, *n* (%)	3,821 (37.8)	853 (33.8)	992 (39.2)	1,007 (39.8)	969 (38.3)	<0.001
Lipid-lowering medication, *n* (%)	1,980 (19.6)	507 (20.1)	518 (20.5)	500 (19.8)	455 (18.0)	0.124

AIP, atherogenic index of plasma; ALT, alanine aminotransferase; AST, aspartate aminotransferase; BMI, body mass index; BUN, blood urea nitrogen; DBP, diastolic blood pressure; DKD, diabetic kidney disease; FBG, fasting blood glucose; FCp, fasting serum C-peptide; HbA1c, glycated hemoglobin; HDL-C, high-density lipoprotein cholesterol; LDL-C, low-density lipoprotein cholesterol; PBG, postprandial blood glucose; PCp, postprandial serum C-peptide; Q, quartile; SBP, systolic blood pressure; Scr, serum creatinine; TC, total cholesterol; TG, triglycerides; UA, uric acid.

### 3.2 Relationship between AIP and DKD in T2DM

**Figure 2:**
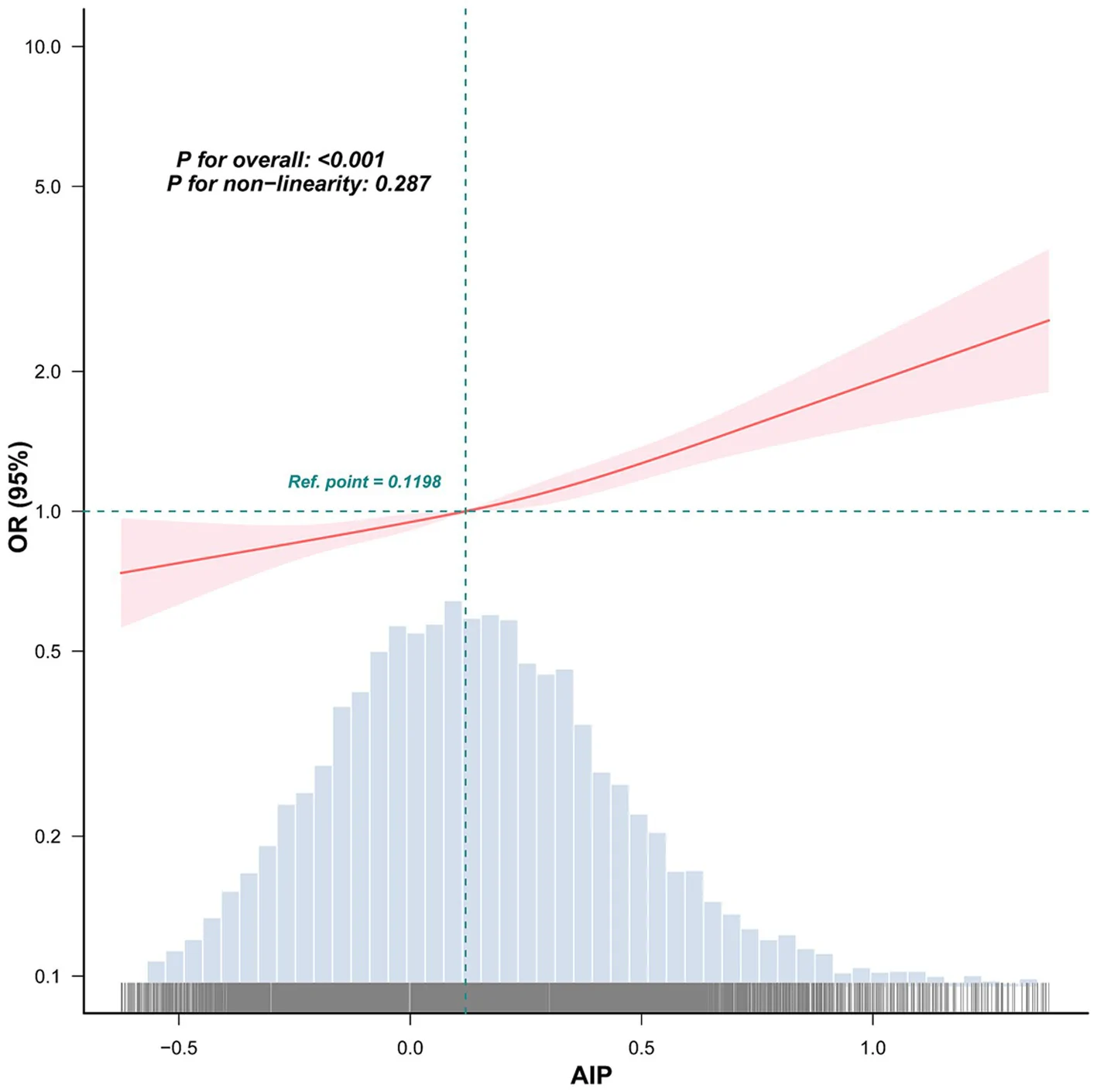
Dose–response relationship between AIP and DKD. Restricted cubic spline regression with adjustment for age, sex, blood pressure, glycemic parameters, body mass index, fasting C-peptide, and lipid-lowering and antihypertensive medications. The solid line represents the estimated OR, and the shaded area represents the 95% confidence interval. The histogram at the bottom shows the distribution of AIP values.

RCS analysis indicated a positive association between AIP and DKD prevalence with rising AIP levels after adjustment for all covariates (Figure 2). Relationships were linear (P for non-linearity >0.05), indicating a steady escalation in risk across the AIP spectrum.

The multivariable logistic regression results indicated a marked positive relationship between AIP and DKD. In the unadjusted model (Model 1), each one-unit increase in AIP corresponded to a 90% higher odds of DKD (OR: 1.90, 95% CI: 1.70–2.14, *p* < 0.001). This relationship remained stable after sequential adjustment for demographic, clinical, and treatment-related variables (Models 2–4). In Model 4, each one-unit AIP rise corresponded to a 72% greater likelihood of prevalence of DKD (OR: 1.72, 95% CI: 1.51–1.96, *p* < 0.001).

Analysis of AIP as a categorical variable showed the presence of a clear dose–response gradient in all quartiles (P for trend <0.001). Compared to Q1, individuals in Q3 showed a 27% greater likelihood of DKD (OR: 1.27, 95% CI: 1.12–1.43, *p* < 0.001), while those in Q4 had a 54% increase (OR: 1.54, 95% CI: 1.36–1.75, *p* < 0.001) in Model 4. In Q2, the relationship decreased to non-significance following full adjustment (OR: 1.10, 95% CI: 0.98–1.24, *p* = 0.119). Details are provided in Table 2.

**Table 2:** Multivariable logistic regression analysis of the association between AIP and DKD.

Variable	Model 1		Model 2		Model 3		Model 4	
	OR (95% CI)	*P*-value	OR (95% CI)	*P*-value	OR (95% CI)	*P*-value	OR (95% CI)	*P*-value
AIP (continuous)	1.9 (1.7 ~ 2.14)	<0.001	2.33 (2.07 ~ 2.63)	<0.001	1.74 (1.53 ~ 1.99)	<0.001	1.72 (1.51 ~ 1.96)	<0.001
AIP (categories)								
Q1	1 (Ref)		1 (Ref)		1 (Ref)		1 (Ref)	
Q2	1.16 (1.04 ~ 1.3)	0.008	1.2 (1.07 ~ 1.35)	0.001	1.11 (0.99 ~ 1.25)	0.075	1.1 (0.98 ~ 1.24)	0.119
Q3	1.36 (1.22 ~ 1.52)	<0.001	1.45 (1.29 ~ 1.62)	<0.001	1.29 (1.14 ~ 1.45)	<0.001	1.27 (1.12 ~ 1.43)	<0.001
Q4	1.74 (1.55 ~ 1.94)	<0.001	2.04 (1.81 ~ 2.29)	<0.001	1.57 (1.39 ~ 1.78)	<0.001	1.54 (1.36 ~ 1.75)	<0.001
P for trend		<0.001		<0.001		<0.001		<0.001

Model 1: Crude model (unadjusted); Model 2: Adjusted for age and sex; Model 3: Adjusted for age, sex, DBP, SBP, FBG, HbA1c, and FCp; Model 4: Adjusted for age, sex, DBP, SBP, FBG, HbA1c, FCp, BMI, lipid-lowering medication, and antihypertensive medication. AIP, atherogenic index of plasma; CI, confidence interval; DKD, diabetic kidney disease; OR, odds ratio; Q, quartile.

External validation in the NHANES cohort (*n* = 5,573; DKD prevalence: 39.2%) confirmed the AIP-DKD association (Supplementary Table 3). In the crude model, each 1-unit increment in AIP was associated with a 50% higher odds of DKD (OR: 1.50, 95% CI: 1.28–1.76, *p* < 0.001), which remained significant after multivariable adjustment (OR: 1.35, 95% CI: 1.10–1.65, *p* = 0.003). In quartile analyses, compared with the lowest quartile (Q1), the adjusted odds ratios for Q2, Q3, and Q4 were 0.93 (95% CI: 0.77–1.12, *p* = 0.443), 0.97 (95% CI: 0.81–1.17, *p* = 0.781), and 1.27 (95% CI: 1.05–1.53, *p* = 0.013), respectively. A significant dose–response trend was observed (P for trend = 0.012). Overall, these findings align closely with those from the primary discovery cohort, supporting the robustness and generalizability of our main results.

### 3.3 Subgroup analysis

**Figure 3:**
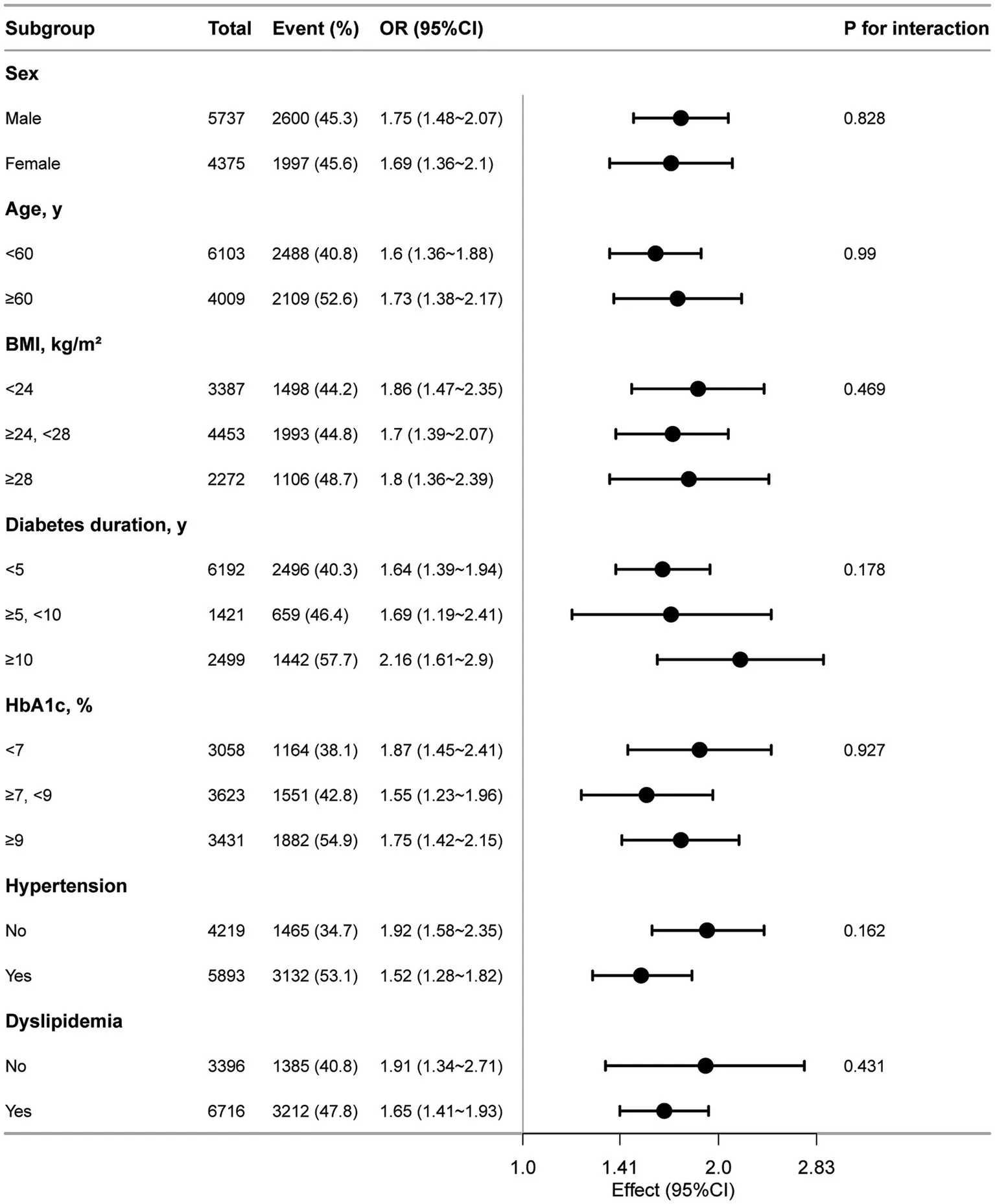
Subgroup analysis of the association between AIP and DKD. Estimates were derived from multivariable logistic regression models adjusted for age, sex, DBP, SBP, FBG, HbA1c, FCp, BMI, lipid-lowering medication, and antihypertensive medication, with the stratification variable excluded from the respective model.

Subgroups were analyzed to examine the influence of clinically relevant stratification on the AIP-DKD relationship (Figure 3). The results indicated that sex, age, BMI, diabetes duration, HbA1c, hypertension and dyslipidemia had no marked influence on the relationship, with an absence of significant interactions (all P for interaction > 0.05).

### 3.4 Discriminative performance

AUC, NRI and IDI were calculated to assess whether AIP improved DKD discrimination beyond established risk factors (Table 3). The baseline model, which included covariates from the model 4, yielded an AUC of 68.42% (95% CI: 67.39–69.45%). After incorporating AIP, the AUC increased modestly to 68.76% (95% CI: 67.74–69.80%). Although the addition of AIP to the baseline model resulted in a statistically significant improvement in AUC as assessed by DeLong test (*p* < 0.05), the absolute increment was only 0.34%. Further evaluation using reclassification metrics demonstrated that adding AIP significantly enhanced predictive performance, with a continuous NRI of 0.118 (95% CI: 0.079–0.157, *p* < 0.001) and an IDI of 0.006 (95% CI: 0.004–0.007, *p* < 0.001).

**Table 3:** Evaluation of the incremental predictive utility of AIP and related indices.

Model	C-statistic (95%CI)	*P* value	NRI (continuous) (95%CI)	*P* value	IDI (95%CI)	*P* value
Basic model	0.684 (0.674, 0.694)	Ref	Ref	Ref	Ref	Ref
Basic model+AIP	0.688 (0.677, 0.698)	=0.015	0.118 (0.079, 0.157)	<0.001	0.006 (0.004, 0.007)	<0.001

Basic model adjustment included: age, sex, DBP, SBP, FBG, HbA1c, FCp, BMI, lipid-lowering medication, and antihypertensive medication.

## 4 Discussion

This large cross-sectional analysis of the real-world hospital cohort and the nationally representative NHANES database observed a marked and independent positive link between AIP and DKD. This relationship persisted after extensive adjustments for demographic variables, metabolic factors, and medications. Notably, the association followed a linear dose–response pattern and remained intact throughout multiple clinically relevant subgroups. Furthermore, incorporating AIP into conventional risk models led to modest but statistically significant improvements in discriminative performance, as evidenced by increases in AUC, NRI, and IDI.

Our findings are in agreement with of literature demonstrating a positive link between AIP and DKD in diabetic populations. A meta-analysis involving 25,773 participants reported a pooled risk ratio of 1.51 for DKD associated with elevated AIP levels (20). Prospective data from a Chinese cohort of 2,943 individuals further showed that both higher baseline AIP and persistently elevated AIP trajectories were independently associated with incident DKD (14). Similar associations have been described by Zhang et al. (21), Li et al. (12), and Yadegar et al. (22) in various populations in Iran and China. Evidence also supports a role for AIP in early renal injury, with predictive value demonstrated for microalbuminuria in newly diagnosed patients (16). Analyses of NHANES data have likewise identified strong relationships between AIP and albuminuria or chronic kidney disease in individuals with diabetes (8, 23). Although one study did not observe a significant association with reduced eGFR (8), additional work by Liu et al. (24) confirmed that elevated AIP predicts accelerated decline in renal function in a Chinese population.

Despite overall consistency, some differences between our results and previous studies merit consideration. In contrast to reports from two large NHANES-based analyses that described a U-shaped relationship between CKD and AIP in general populations (25, 26), our study demonstrated a linear positive association. This discrepancy likely reflects differences in study populations, as our cohort consisted exclusively of patients with T2DM, in whom metabolic disturbances may exert more uniform effects on renal risk. Additionally, while one prior study suggested that the AIP-CKD association was significant only in men (25), our subgroup analyses did not reveal sex-specific differences, possibly due to differences in outcome definitions (CKD vs. DKD) and population characteristics. Moreover, a few smaller studies conducted in Chinese cohorts failed to detect a significant link between AIP and renal dysfunction (15, 17). These null findings are likely attributable to limited sample sizes and insufficient adjustment for important confounders, such as lipid-lowering therapy, rather than indicating a true absence of association.

Based on published basic research and preclinical study data, the association between elevated AIP and DKD is likely driven by a combination of interrelated biological processes, including lipid-induced toxicity, oxidative injury, hemodynamic disturbances, and vascular endothelial dysfunction. Higher AIP values are a reflection of unfavorable lipid profiles involving increased TG-rich lipoproteins and decreased HDL cholesterol. This imbalance facilitates lipid deposition within both glomerular and tubular compartments, promoting mitochondrial impairment, endoplasmic reticulum stress, and programmed cell death, which are key features of progressive renal damage (27, 28). PTECs are particularly susceptible to lipotoxicity given their high oxidative phosphorylation demand; lipid accumulation in these cells drives inflammation and fibrosis via TNF-*α*/IL-6/TGF-β1 signaling (29). At the same time, oxidized lipid species can activate pro-inflammatory signals, including the NF-κB axis, in intrinsic renal cells, leading to cytokine production, extracellular matrix accumulation, and fibrotic remodeling (30–32). In the hypoxic diabetic kidney, HIF-1α stabilization shifts tubular metabolism from fatty acid oxidation to glycolysis, generating excessive ROS and promoting EMT and fibrosis (33, 34). Tubular injury biomarkers (KIM-1, NGAL, β2-MG) accordingly emerge as sensitive early predictors of DKD (29). Insulin resistance, which frequently accompanies elevated AIP, further contributes to glomerular hyperfiltration and renin-angiotensin-aldosterone system activation, thereby driving accelerated albuminuria and nephron loss (35–37). In addition, atherogenic dyslipidemia compromises endothelial nitric oxide availability and increases glomerular capillary permeability, allowing lipoproteins to infiltrate the glomerular tuft and induce podocyte injury and detachment, both of which are critical steps in the development of albuminuria (38, 39). Notably, cardiovascular factors, including aortic and valvular calcification, LVH, and elevated LVMI, are independent predictors of renal and cardiovascular endpoints in DKD, highlighting the cardiorenal axis (40). Although the cross-sectional design of this study precludes causal inference, these previously established mechanisms offer a plausible biological basis that may account for the observed statistical association between AIP and DKD. Future experimental studies are needed to directly test these mechanistic hypotheses.

This article has several important strengths and provides clinically relevant insights, although certain limitations should also be acknowledged. A key strength is the large and well-defined cohort of 10,112 individuals with T2DM, which ensures sufficient power to detect modest associations and supports detailed subgroup analyses across age groups, body mass index categories, and diabetes duration. In contrast to many prior studies relying on less standardized data collection, our investigation utilized the structured framework of the National Metabolic Management Center, ensuring consistency in laboratory measurements and clinical assessments and thereby reducing information bias. From an analytical standpoint, we employed sequential multivariable models to rigorously evaluate the independent relationship between AIP and DKD. The use of RCS modeling allowed us to characterize the dose–response relationship flexibly, without assuming linearity *a priori*. Furthermore, the stability of the link was assessed across clinically relevant strata by subgroup and interaction analyses, and reclassification metrics including NRI and IDI were applied for quantification of the incremental predictive utility of AIP apart from that afforded by conventional risk factors. In addition, it should be objectively noted that although the improvement in AUC after adding AIP to the model reached statistical significance (*p* < 0.05), the absolute increase was only 0.34% (from 68.42 to 68.76%). Given the large sample size of over 10,000 cases, this marginal improvement readily achieved statistical significance, yet it suggests that the incremental predictive value of AIP as an independent screening or adjunctive diagnostic tool in clinical practice is extremely limited. Future studies should explore combination strategies of AIP with other biomarkers, or reassess its clinical utility through prospective designs. Collectively, these methodological approaches strengthen the internal validity of our findings and provide a more nuanced understanding of the AIP-DKD relationship. In a clinical context, AIP is derived from routine lipid parameters that are inexpensive and widely accessible, rendering it a useful means of assessing risk in everyday practice, particularly in primary care or resource-limited settings. To explore the stability of the AIP-DKD association and to assess potential effect modification, we performed subgroup analyses stratified by clinically relevant factors. The choice of cutoff points was guided by established clinical criteria: Age was stratified at 60 years, a cutoff commonly used in Chinese diabetes guidelines to define elderly diabetes. BMI was categorized according to the Chinese Working Group on Obesity criteria: < 24 kg/m^2^ (normal), 24–< 28 kg/m^2^ (overweight), and ≥ 28 kg/m^2^ (obesity). Diabetes duration was stratified at 5 and 10 years: 5 years marks the threshold for increased microvascular complication risk and screening initiation, while 10 years distinguishes long-standing diabetes with substantially higher DKD prevalence. HbA1c was classified at 7 and 9%, representing the general glycemic target and the threshold for poor control requiring treatment intensification, respectively, allowing assessment across three glycemic control tiers. Across all these strata, the AIP-DKD association remained consistent, with no significant interactions detected. This homogeneity across diverse patient subgroups enhances the generalizability of our findings. Although causality cannot be inferred, the consistent linear association observed across multiple subgroups suggests that AIP may have utility in the identification of individuals with T2DM at elevated risk of renal complications. Incorporating AIP into routine annual evaluations, alongside established measures of kidney function, may facilitate earlier identification of high-risk individuals, thereby enabling appropriate intervention. Future longitudinal investigations are requires to verify the temporal association and to determine whether interventions targeting AIP such as lifestyle modification or lipid-lowering therapy can effectively delay or prevent DKD progression.

Several limitations warrant acknowledgement. First, the study was cross-sectional, thereby precluding causal inference, and further prospective investigations are needed for verification. Second, certain potential confounding factors, including diet, family history, and physical activity, were not available in the dataset, leaving the possibility of residual confounding despite comprehensive adjustment. Third, although the primary analysis was single-center, external validation using the nationally representative NHANES cohort confirmed the consistency of our findings, supporting their generalizability. However, further validation in additional diverse cohorts is still needed to confirm the robustness of our results across different populations. Furthermore, the diagnosis of diabetic kidney disease in this study relied on a single measurement of UACR or estimated glomerular filtration rate (eGFR) and did not include confirmation of persistent microalbuminuria by repeat testing over 3–6 months. A single UACR measurement is vulnerable to transient factors such as infection, fever, strenuous exercise, and fluctuations in blood glucose and blood pressure, which may introduce measurement bias and lead to false-positive diagnoses. Accordingly, this study may overestimate the prevalence of DKD and the magnitude of its association with AIP. Future research should adopt a prospective design with repeat assessments of renal function and albuminuria to validate these findings. Finally, a single measurement was used for AIP calculation, which may not fully capture the long-term effects of lipid levels. As such, repeated assessments would be valuable for evaluating temporal variability and its relationship with renal outcomes.

## 5 Conclusion

In summary, this cross-sectional analysis demonstrates that AIP is independently associated with DKD prevalence and shows a linear dose–response relationship. The association is consistent across a range of clinical subgroups, and AIP provides incremental discriminative value beyond traditional risk factors, however, the improvement in discriminative ability for DKD was modest. Given its accessibility and low cost, AIP may have utility in determining individuals with DKD.

## Data Availability

The original contributions presented in the study are included in the article/Supplementary material, further inquiries can be directed to the corresponding authors.
