# Supplementary material for: Linear association between atherogenic index of plasma and diabetic kidney disease: evidence from a large clinical cohort and NHANES
Source: Front Nutr. 2026 Aug 11;13:1853881. doi: 10.3389/fnut.2026.1853881 (PMC13503233; doi:10.3389/fnut.2026.1853881)
Supplement: Supplementary file 1 [file Table_1.docx]

**Supplementary Table 1 Missing data and miss rates of covariates**

| **Characteristics** | **Total (n = 10,112)** |
| --- | --- |
| Sex, n (%) | 0 (0.0%) |
| Age, years | 0 (0.0%) |
| Education level, n (%) | 4 (0.0%) |
| BMI, kg/m² | 3 (0.0%) |
| SBP, mm Hg | 2 (0.0%) |
| DBP, mm Hg | 2 (0.0%) |
| Diabetes duration, years | 0 (0.0%) |
| FBG, mmol/L | 2 (0.0%) |
| PBG, mmol/L | 149 (1.5%) |
| HbA1c, % | 25 (0.2%) |
| FCp, ng/mL | 102 (1.0%) |
| PCp, ng/mL | 160 (1.6%) |
| TG, mmol/L | 0 (0.0%) |
| TC, mmol/L | 0 (0.0%) |
| HDL-C, mmol/L | 0 (0.0%) |
| LDL-C, mmol/L | 0 (0.0%) |
| BUN, mmol/L | 24 (0.2%) |
| Scr, μmol/L | 22 (0.2%) |
| UA, μmol/L | 27 (0.3%) |
| ALT, U/L | 54 (0.5%) |
| AST, U/L | 58 (0.6%) |
| Hypertension, n (%) | 0 (0.0%) |
| Dyslipidemia, n (%) | 0 (0.0%) |
| Smoking, n (%) | 0 (0.0%) |
| Alcohol consumption, n (%) | 0 (0.0%) |
| Antihypertensive medication, n (%) | 0 (0.0%) |
| Lipid-lowering medication, n (%) | 0 (0.0%) |

All abbreviations are listed in Table 1.

**Supplementary Table 2 The variance inflation factor (VIF) of covariates.**

| Variables | Variance inflation factor (VIF) |
| --- | --- |
| Crude | 1.183 |
| Sex | 1.112 |
| Age | 1.557 |
| DBP | 2.098 |
| SBP | 1.843 |
| FBG | 1.866 |
| HbA1c | 1.913 |
| FCp | 1.168 |
| BMI | 1.242 |
| Lipid-lowering medication | 1.051 |
| Antihypertensive medication | 1.165 |

**Supplementary Table 3 Multivariable logistic regression analysis of the association between AIP and DKD**

| **Variable** | **N** | **DKD (%)** | **Crude model** | | **Adjusted Model** | |
| --- | --- | --- | --- | --- | --- | --- |
|  |  |  | **OR (95%CI)** | **P value** | **OR (95%CI)** | **P value** |
| AIP  (continuous) | 5573 | 2182 (39.2) | 1.5 (1.28~1.76) | <0.001 | 1.35 (1.1~1.65) | 0.003 |
| AIP  (categories) |  |  |  |  |  |  |
| Q1 | 1390 | 501 (36) | 1(Ref) |  | 1(Ref) |  |
| Q2 | 1396 | 518 (37.1) | 1.05 (0.9~1.22) | 0.56 | 0.93 (0.77~1.12) | 0.443 |
| Q3 | 1393 | 550 (39.5) | 1.16 (0.99~1.35) | 0.061 | 0.97 (0.81~1.17) | 0.781 |
| Q4 | 1394 | 613 (44) | 1.39 (1.2~1.62) | <0.001 | 1.27 (1.05~1.53) | 0.013 |
| P for trend | 5573 | 2182 (39.2) |  | <0.001 |  | 0.012 |

Adjusted Model: adjusted for age, sex, DBP, SBP, FBG, HbA1c, BMI, lipid-lowering medication, and antihypertensive medication.
